# Honokiol-Loaded β-D-Glucan/Chitosan Nanoparticles with Antibacterial and Anti-Inflammatory Activities for Promoting MRSA-Infected Wound Healing

**DOI:** 10.3390/pharmaceutics18091184

**Published:** 2026-09-19

**Authors:** Shouli Yi, Ming Guan, Hongjuan Zhang, Ping Qiu, Shengyi Wang, Tingjun Hu

**Affiliations:** 1 College of Animal Science and Technology, Guangxi University, Nanning 530005, China; yishouli@st.gxu.edu.cn (S.Y.); 2134130102@st.gxu.edu.cn (M.G.); 2 Key Laboratory of New Animal Drug Project, Gansu Province, Key Laboratory of Veterinary Pharmaceutical Development, Ministry of Agriculture and Rural Affairs, Lanzhou Institute of Husbandry and Pharmaceutical Sciences of Chinese Academy of Agriculture Sciences, Lanzhou 730050, China; zhanghongjuan@caas.cn (H.Z.); 821012450925@caas.cn (P.Q.)

**Keywords:** antibacterial, MRSA, honokiol, β-D-glucan, wound healing

## Abstract

**Background:** Methicillin-resistant *Staphylococcus aureus* (MRSA) wound infections are a major clinical concern due to their high prevalence and the growing risk of antimicrobial resistance. Therefore, it is imperative to develop safe and effective therapeutic strategies. **Methods**: Herein, we fabricated a novel antibiotic-free nanodrug delivery system (HNK/DG-COS NPs) by loading honokiol (HNK), a hydrophobic natural antimicrobial agent, onto a dual-polysaccharide carrier composed of chitosan (COS) and β-D-glucan (DG), for the treatment of MRSA-infected wounds. COS endows the nanoparticles with synergistic antibacterial activity together with HNK, while DG improves their anti-inflammatory and tissue repair capacities. The physicochemical characteristics of nanoparticles, as well as their in vitro antibacterial and anti-inflammatory activities, were systematically evaluated. Furthermore, an MRSA-infected wound model was constructed to verify their therapeutic efficacy. **Results**: HNK/DG-COS NPs exhibited a spherical morphology with a uniform particle size of approximately 200 nm. In vitro experiments have shown that HNK/DG-COS NPs can significantly kill MRSA and inhibit biofilm formation. Scanning electron microscopy further revealed that the nanoparticles exerted antibacterial effects by disrupting the integrity of bacterial cell membranes. In LPS-stimulated RAW264.7 macrophages, treatment with HNK/DG-COS NPs significantly mitigated pro-inflammatory cytokine levels. Cell migration assays confirmed that nanoparticles effectively enhanced L929 cell migration. In vivo, treatment with HNK/DG-COS NPs significantly accelerated MRSA-infected wound healing in mice, mitigated inflammatory damage, stimulated collagen production and repaired damaged tissues. **Conclusions**: HNK/DG-COS NPs represent a promising antibiotic-free candidate for intervention against early-stage MRSA-infected wounds.

## 1. Introduction

At present, bacterial infections still account for a large proportion of global morbidity and mortality. Among them, methicillin-resistant *Staphylococcus aureus* (MRSA) is one of the most prevalent pathogens causing wound infections [1,2]. After colonizing skin wounds, MRSA can easily form dense biofilms, which continuously lead to delayed wound healing and deep invasive infections. Dysregulated inflammation is another key refractory pathological characteristic of MRSA-infected wounds, further impeding the healing progression of wounds [3,4]. Currently, antibiotics remain the primary treatment for MRSA-infected wounds, but due to the overuse of antibiotics, there is an accompanying increase in antibiotic resistance and severe side effects. Therefore, developing new therapeutic strategies for MRSA-infected wounds is urgently required.

Phytochemicals derived from traditional Chinese medicines exert diverse pharmacological functions, including bactericidal, anti-inflammatory, antioxidant and tissue regenerative effects, and are capable of modulating multiple pathological stages of MRSA-infected wounds, thus attracting extensive research attention in wound infection therapy in recent years. Honokiol (HNK) is a polyphenolic compound extracted from the traditional Chinese medicine *Magnolia officinalis*, and it exerts multiple effects on various metabolic pathways and physiological functions [5]. Notably, HNK can effectively inhibit the growth of both Gram-negative and Gram-positive bacteria, as well as the formation of biofilms. In addition, it eliminates bacteria embedded in biofilms and suppresses bacterial motility [6,7,8]. However, the problems of low water solubility, poor bioavailability and inadequate skin penetration of HNK severely limit its clinical application.

Over the past few decades, advances in nanotechnology have provided a promising approach for the translational applications of hydrophobic antimicrobial agents, as nanoscale drug delivery systems can improve the aqueous solubility, stability, bioavailability and even transdermal penetration of such drugs [9]. Many nanomedicines have been developed and achieved the desired antibacterial effect without drug resistance [10]. However, diverse antibacterial nanoplatforms still have the problems of limited biodegradability, severe adverse effects and high preparation costs. More importantly, various nanoparticles cannot achieve adequate control of the pathological processes in wound infections, including bacterial and biofilm infections, refractory inflammation, and impaired tissue repair. Hence, developing multifunctional, green, and sustainable nanoplatforms that orchestrate the entire wound-healing cascade remains a critical yet unmet challenge in this field.

Chitosan (COS) is a natural cationic polysaccharide with favorable biocompatibility, biodegradability and antibacterial activity [11]. Its antibacterial effect is mainly attributed to electrostatic interactions between its protonated amino groups (-NH_3_^+^) and negatively charged bacterial membranes. Such interactions increase bacterial membrane permeability, trigger the leakage of intracellular contents, and ultimately lead to bacterial lysis [12,13]. As a promising natural antibacterial material, COS has been extensively utilized in biomedicine and other related fields [14,15,16,17,18]. β-D-Glucan (DG), another natural polysaccharide, has also gained widespread interest in the preparation of wound-healing carriers, owing to its attractive anti-inflammatory and immunomodulatory properties, as well as its capacity to accelerate skin cell regeneration [19]. Therefore, the combination of COS and DG represents a potential strategy for constructing a nanocarrier to treat MRSA-infected wounds. By leveraging the antibacterial activity of COS together with the anti-inflammatory and tissue regeneration capacity of DG, this nanoplatform can achieve comprehensive control of the full pathological processes in MRSA-infected wounds.

**Scheme 1:**
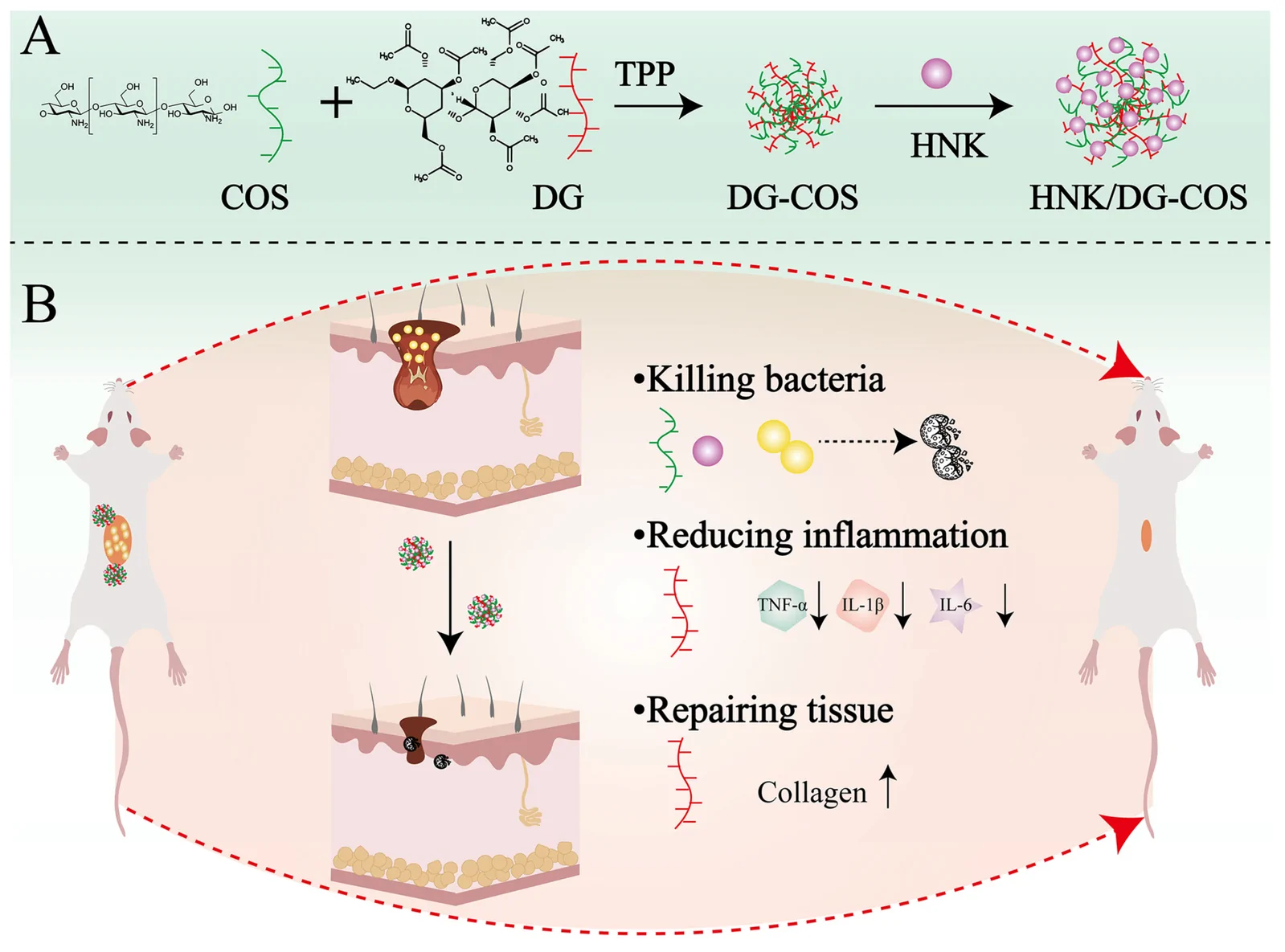
Schematic illustration of the preparation and therapeutic mechanism of HNK/DG-COS NPs. (**A**) Preparation method of HNK/DG-COS NPs. (**B**) HNK/DG-COS NPs promote MRSA-infected skin wound healing.

In this study, we developed novel HNK nanoparticles based on two natural polysaccharides, COS and DG. COS endows the nanoparticles with synergistic antibacterial effects together with honokiol, while DG enhances their anti-inflammatory and tissue repair capacities. This work not only offers a green, degradable, and biocompatible functional carrier for the hydrophobic natural antimicrobial agent HNK but also provides a safe therapeutic strategy for the clinical treatment of MRSA-infected wounds (Scheme 1).

## 2. Materials and Methods

### 2.1. Materials

Honokiol was purchased from Shanghai Macklin Biochemical Technology Co., Ltd. (Shanghai, China). Chitosan (catalog No. C105799; degree of deacetylation ≥ 95%) was purchased from Shanghai Aladdin Biochemical Technology Co., Ltd. (Shanghai, China). β-D-Glucan (DG; from barley; catalog No. G6513; purity ≥ 95%) was purchased from Sigma-Aldrich (Shanghai, China). A CCK-8 assay kit was obtained from BioSharp (Beijing Langjieko Technology Co., Ltd., Beijing, China). Crystal violet and propidium iodide (PI) were obtained from Servicebio (Wuhan Servicebio Technology Co., Ltd., Wuhan, China). ELISA kits were obtained from Melian Biotechnology Co., Ltd. (Shanghai, China) (Enzyme-linked Biotechnology Co., Ltd. (Shanghai, China)). DMEM medium and fetal bovine serum were obtained from Gibco (Thermo Fisher Scientific, Waltham, MA, USA). MH Broth, Mueller-Hinton Agar, TSA Agar, and Trypticase (Tryptic) Soy Broth were provided by Qingdao Hope Bio-Technology Co., Ltd. (Qingdao, China). MRSA (ATCC 43300) was bought from the American Type Culture Collection (Manassas, VA, USA).

### 2.2. Preparation and Characterization of DG-COS NPs and HNK/DG-COS NPs

β-D-Glucan was dissolved in deionized water at a concentration of 100 μg/mL under continuous stirring at 50 °C for 30 min. Chitosan was dissolved in 1% (*v*/*v*) aqueous acetic acid solution at a concentration of 100 μg/mL, and the pH was adjusted to 5.0–5.5 using 1 M NaOH. Both solutions were filtered through a 0.22 μm syringe filter prior (Solarbio, Beijing, China) to use. For the preparation of DG-COS NPs, the DG solution (1.5 mL) was slowly added into the COS solution (1.5 mL) under vigorous magnetic stirring (800 r/min) at room temperature. The mixture was then stirred for an additional 2 h to allow complete electrostatic complexation. Subsequently, 300 μL of tripolyphosphate (TPP, 1 mg/mL) was added dropwise to the mixture under continued stirring, and the reaction was allowed to proceed for another 12 h at room temperature to induce ionic crosslinking. The resulting suspension was transferred to a dialysis bag (MWCO: 3500 Da) and dialyzed against deionized water for 24 h to remove unreacted TPP, acetic acid, and other small molecule impurities, yielding DG-COS NPs. Honokiol-loaded DG-COS NPs (HNK/DG-COS NPs) were prepared following the same procedure, except that 20 μL of honokiol solution (1 mg/mL in methanol) was added to the COS solution and stirred for 30 min prior to the dropwise addition of DG solution, ensuring sufficient incorporation of honokiol before nanoparticle assembly. The subsequent TPP addition, crosslinking, and dialysis steps were identical to those described above.

The morphology of DG-COS NPs and HNK/DG-COS NPs was observed using transmission electron microscopy (TEM; JEOL JEM-F200, JEOL Ltd., Tokyo, Japan), and their particle size and zeta potential were measured using a Malvern Zetasizer Nano (Malvern Panalytical, Malvern, UK). In addition, X-ray diffraction (XRD), Raman spectroscopy and Fourier-transform infrared spectroscopy (FTIR) analyses were performed on HNK, DG, COS, DG-COS NPs and HNK/DG-COS NPs. HNK/DG-COS NPs were stored at 4 °C for 21 days, and the particle size was monitored to evaluate their stability.

### 2.3. In Vitro Antibacterial Activity

MRSA at a concentration of 1 × 10^8^ CFU/mL was co-incubated with HNK, DG, COS, DG-COS NPs and HNK/DG-COS NPs for 12 h. The bacterial suspension was then diluted and spread onto agar plates, which were incubated at 37 °C for 16 h. Bacterial viability was assessed by the standard plate count method, and the antibacterial inhibition rate was calculated as the percentage reduction in colony-forming units (CFUs) in the treatment group relative to the control group. Samples were taken from the DG-COS NP and HNK/DG-COS NP groups at 0, 2, 4, 6, 8, 10, 12, 24 and 48 h and spread onto plates to evaluate the antibacterial efficacy at different time points [20,21].

### 2.4. Live/Dead Staining of Bacteria

The treated bacteria were collected by centrifugation, rinsed with saline, and then resuspended to a final concentration of 1 × 10^8^ CFU/mL. Staining working solution (1 μL per 100 μL suspension) was added and incubated at 37 °C for 15 min in the dark and then observed under fluorescence microscopy [22].

### 2.5. SEM Analysis

The treated bacteria were fixed in 2.5% glutaraldehyde at 4 °C overnight, then dehydrated in graded ethanol (30–100%), dried, and examined by scanning electron microscopy (SEM; Hitachi SU8010, Hitachi, Tokyo, Japan) [23].

### 2.6. Bacterial Membrane Rupture

The treated bacteria were washed thrice with 5 mM HEPES, resuspended in a black 96-well plate, and incubated with PI (7.5 μg/mL) at 37 °C for 30 min; the fluorescence intensity was measured using a microplate reader (excitation, 535 nm; emission, 615 nm). The supernatant was collected to determine the concentration of polysaccharides, proteins and eDNA.

### 2.7. Biofilm Inhibition

Preliminary evaluation of the ability to inhibit biofilm formation was conducted using the crystal violet staining method [24,25]. A suspension of 1 × 10^8^ CFU/mL MRSA was inoculated onto 24-well plates containing HNK, DG, COS, DG-COS NPs and HNK/DG-COS NPs, and incubated at 37 °C for 24 h. Planktonic bacteria were gently washed away with distilled water, fixed in ethanol for 15 min, stained with 0.1% crystal violet for 10 min, and decolorized with 33% acetic acid before absorbance was measured at 570 nm.

Concurrently, three-dimensional reconstruction was performed using a confocal laser scanning microscope following SYTO-9/PI staining. Furthermore, the biofilm was washed with saline to obtain an eluate suspension; after sonication and centrifugation, the supernatant was filtered through a 0.22 μm filter membrane, and the contents of extracellular polysaccharides, proteins and eDNA in the supernatant were determined [26,27].

### 2.8. Cell Migration Assay

L929 cells (1 × 10^6^ cells/mL) were seeded into a 24-well plate. When confluence reached 95%, a scratch of uniform width was drawn, and detached cells were gently wash away with PBS. For the control group, the medium was replaced with low-serum medium. The appropriate drug was added to the remaining groups. The scratches were photographed at 0, 24 and 48 h, and cell migration ability was evaluated based on the width of the scratch.

### 2.9. Anti-Inflammatory Assay

RAW264.7 macrophages were seeded in a 24-well plate (1 × 10^6^ cells per well). Once confluence reached over 90%, 1 μg/mL LPS was added to stimulate an inflammatory response for 12 h. Subsequently, various drugs were added and co-incubated for 12 h; the supernatant was then collected, and the concentrations of IL-6, IL-10, IL-1β and TNF-α were measured using an ELISA kit.

### 2.10. CCK-8 Assay

L929 and RAW264.7 cells were seeded into a 96-well plate at a density of 1 × 10^4^ cells per well. When confluence reached over 90%, fresh DMEM medium containing different concentrations of DG-COS NPs and HNK/DG-COS NPs was added. Cell viability was assessed at 6, 12 and 24 h using the CCK-8 assay kit.

### 2.11. Live/Dead Cell Viability Staining

L929 and RAW264.7 cells were seeded in 96-well plates. When confluence reached over 90%, fresh DMEM medium containing the aforementioned drugs was added. After 12 h of culture, the cells were stained using a live/dead cell viability staining kit, and images were captured using an inverted fluorescence microscope.

### 2.12. In Vivo Safety Studies

All animal experiments adhered to the ARRIVE standards and received approval from the Ethics Committee of the Lanzhou Institute of Husbandry and Pharmaceutical Sciences of Chinese Academy of Agricultural Sciences on 13 March 2026 (No. 2026-37).

Female BALB/c mice weighing 20–22 g were administered intraperitoneal injections of HNK, DG, COS, DG-COS NPs and HNK/DG-COS NPs. Seven days later, whole-blood samples were collected for a complete blood count, and samples of the liver, heart, spleen, lungs and kidneys were taken for H&E staining.

### 2.13. Skin Wound Infection in Mice

A mouse MRSA-infected full-thickness skin wound model was established based on a previously reported method with minor modifications [28]. Briefly, female BALB/c mice (18–22 g) were anesthetized with isoflurane. After dorsal hair removal, a standardized full-thickness cutaneous wound with a diameter of 1 cm was created on the dorsal skin of each mouse. Subsequently, 50 μL of MRSA bacterial suspension (1 × 10^8^ CFU/mL) was uniformly inoculated onto the wound surface to construct a stable infected wound model.

At 24 h after bacterial inoculation, all modeled mice were randomly divided into eight groups (*n* = 6), including the uninfected control group, model group, DG-COS NP group, HNK/DG-COS NP group, HNK group, DG group, COS group, and vancomycin (VAN) [29] positive control group. All experimental formulations were freshly prepared with sterile PBS as the vehicle. Each mouse received an in situ injection of 100 μL of the corresponding formulation into the wound site at a standardized dose of 1 mg/kg. Mice in the control and model groups received an equal volume of sterile PBS via the same route. Treatments were administered once daily for 7 consecutive days.

To eliminate subjective observer bias, all wound macroscopic observation, image capture, and quantitative analysis were conducted by independent researchers who were blinded to the animal grouping and treatment allocation.

Wound-healing status was dynamically recorded by daily photography throughout the treatment period. The residual wound area of each sample was quantitatively calculated via ImageJ 1.54f (National Institutes of Health, Bethesda, MD, USA), and the relative wound closure rate was further computed to evaluate wound-healing efficacy. On day 7 after continuous treatment, all experimental mice were euthanized. Wound skin tissues were harvested for histological staining analysis (H&E staining and Masson staining) and quantitative detection of residual bacterial burden in wound tissues. Meanwhile, peripheral blood samples were collected, and serum was isolated to determine the levels of inflammatory cytokines including TNF-α, IL-6, IL-10 and IL-1β using commercial ELISA kits.

### 2.14. Data Analysis

All experimental data in this study were processed and analyzed using GraphPad Prism 10 and Origin 2024 software, and all quantitative results are presented as means ± standard deviation. For longitudinal dynamic wound closure data measured at multiple time points, two-way repeated-measures analysis of variance (ANOVA) was applied for statistical analysis, with treatment group as the between-subject variable and time as the within-subject variable. Tukey’s HSD post hoc test was used for pairwise comparison between every two groups at each time point to correct multiple comparison errors. For static endpoint indicators including wound bacterial burden and inflammatory cytokine levels, one-way ANOVA combined with Tukey’s post hoc test was used for multi-group comparison. Unpaired Student’s *t*-test was adopted for statistical comparison between two independent groups. Statistical significance was defined as: * *p* < 0.05, ** *p* < 0.01, and *** *p* < 0.001.

## 3. Results

### 3.1. Preparation and Characterization

**Figure 1:**
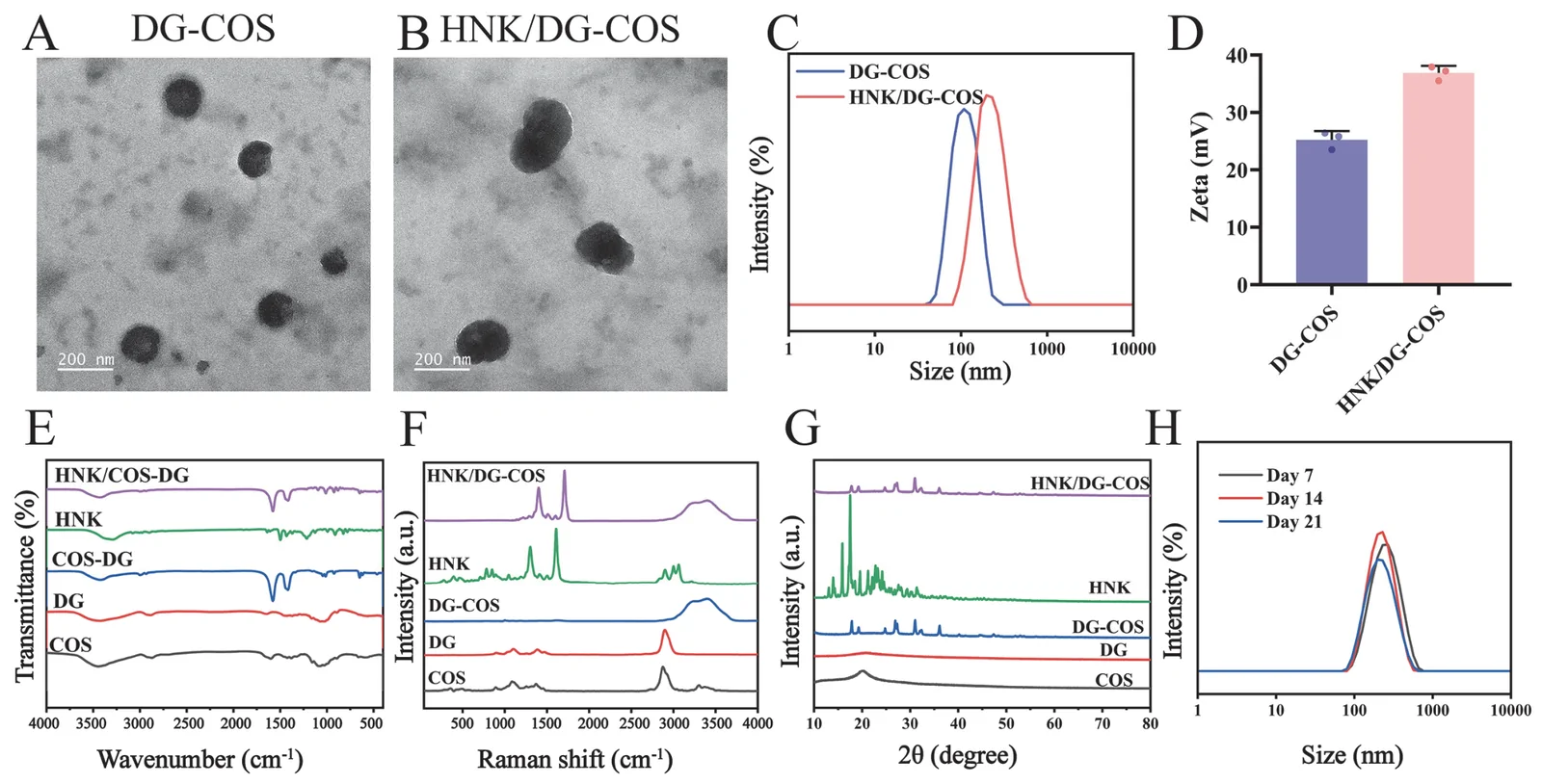
Characterization of DG-COS NPs and HNK/DG-COS NPs. (**A**,**B**) Transmission electron microscopy (TEM) images of DG-COS NPs and HNK/DG-COS NPs. (**C**) Dynamic light scattering (DLS) intensity-based size distribution profile of HNK/DG-COS NPs. (**D**) Zeta potentials of DG-COS NPs and HNK/DG-COS NPs. (**E**) Fourier-transform infrared (FTIR) spectra of DG, COS, HNK, DG-COS NPs, and HNK/DG-COS NPs. (**F**) Raman spectra of DG, COS, HNK, DG-COS NPs, and HNK/DG-COS NPs. (**G**) X-ray diffraction (XRD) patterns of DG, COS, HNK, DG-COS NPs, and HNK/DG-COS NPs. (**H**) Storage stability of HNK/DG-COS NPs over 21 days at 4 °C.

Transmission electron microscopy (TEM) images (Figure 1A,B) showed that both DG-COS NPs and HNK/DG-COS NPs exhibited regular spherical morphology. Dynamic light scattering (DLS) measurements showed that the hydrated particle sizes of DG-COS NPs and HNK/DG-COS NPs were 110.3 ± 2.4 nm and 198.5 ± 3.3 nm, respectively, with zeta potentials of 25.3 ± 0.6 mV and 34.6 ± 1.0 mV and polydispersity indices (PDIs) of 0.167 ± 0.014 and 0.125 ± 0.006, respectively (Figure 1C,D). HPLC analysis revealed an encapsulation efficiency of 90.3 ± 2.2% and a drug loading capacity of 3.2 ± 0.8% for HNK/DG-COS NPs.

Fourier-transform infrared (FTIR) spectroscopy (Figure 1E) showed that DG-COS NPs displayed a new strong peak at 1579 cm^−1^ and the O-H/N-H stretching band shifted from 3440 cm^−1^ to 3424 cm^−1^. After honokiol loading, the FTIR spectrum of HNK/DG-COS NPs exhibited redshifted characteristic peaks of honokiol relative to free honokiol. Raman spectroscopy (Figure 1F) further resolved the O-H/N-H region of DG-COS NPs into a doublet at 3400 and 3266 cm^−1^, while HNK/DG-COS NPs showed honokiol peaks (notably at 1609 and 1302 cm^−1^) redshifted compared with free honokiol.

X-ray diffraction (XRD) patterns (Figure 1G) revealed that chitosan and β-D-glucan were amorphous, whereas DG-COS NPs exhibited several new sharp diffraction peaks at 26.9°, 27.3°, 31.0° and other positions. After loading honokiol, the obtained HNK/DG-COS NPs maintained the main diffraction peaks of DG-COS NPs with slightly decreased intensity, whereas the characteristic crystalline peaks of free honokiol at 15.9°, 17.5° and 22.8° completely disappeared. Storage stability testing (Figure 1H) showed that after 21 days at 4 °C, no significant change in particle size or aggregation was observed for HNK/DG-COS NPs.

### 3.2. In Vitro Anti-MRSA Activity

**Figure 2:**
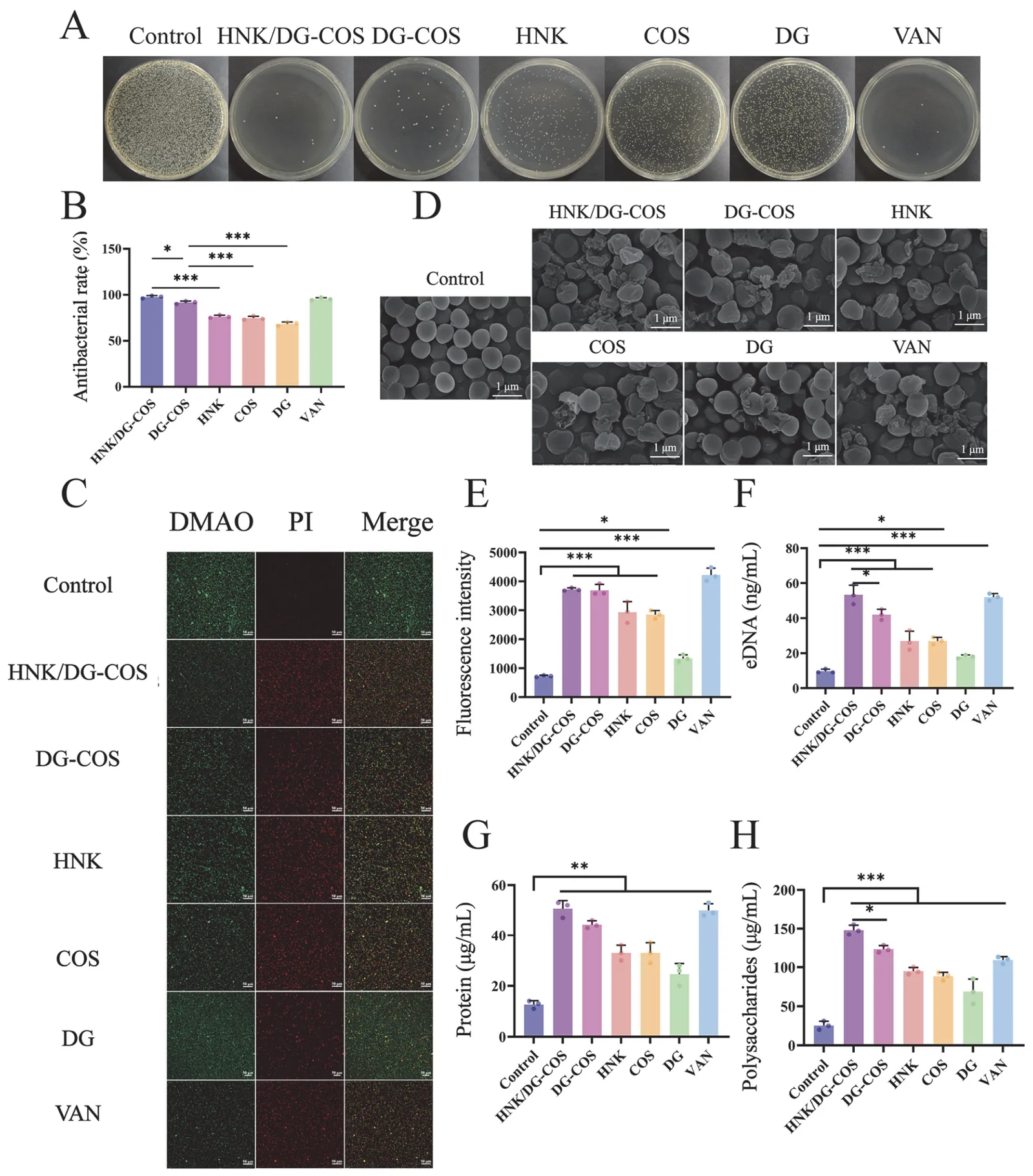
In vitro anti-MRSA activity of HNK/DG-COS NPs. (**A**) Photographs of bacterial colonies on agar plates after MRSA incubation with DG, COS, HNK, DG-COS NPs, and HNK/DG-COS NPs for 12 h. (**B**) Antibacterial rates of DG, COS, HNK, DG-COS NPs, and HNK/DG-COS NPs (*n* = 3). (**C**) Live/dead staining of MRSA after 12 h incubation with DG, COS, HNK, DG-COS NPs, and HNK/DG-COS NPs. Green and red fluorescence indicate live and dead bacteria, respectively. Scale bars: 50 µm. (**D**) Scanning electron microscopy (SEM) images of MRSA after 12 h incubation with DG, COS, HNK, DG-COS NPs, and HNK/DG-COS NPs. Scale bars: 1 µm. (**E**) PI fluorescence intensity after 12 h incubation of MRSA with DG, COS, HNK, DG-COS NPs, and HNK/DG-COS NPs (*n* = 3). (**F**–**H**) Leakage concentrations of extracellular DNA (eDNA), extracellular polysaccharides, and total protein in the supernatant of bacterial cultures following treatment with DG, COS, HNK, DG-COS NPs, and HNK/DG-COS NPs (*n* = 3). Data are presented as means ± SD. One-way ANOVA followed by Tukey’s honest significant difference (HSD) test was used for multiple comparisons. * *p* < 0.05, ** *p* < 0.01, and *** *p* < 0.001.

As shown in Figure 2A,B, both DG-COS NPs and HNK/DG-COS NPs significantly inhibited bacterial growth, and their antibacterial rates were significantly higher than those of the HNK, COS, and DG groups at the same dosage (*p* < 0.001). Among them, the antibacterial rate of HNK/DG-COS NPs was significantly higher than that of DG-COS NPs (*p* < 0.05) (Figure 2B), indicating that the loading of HNK further enhanced the antibacterial efficiency of the nanoparticles. Time-dependent killing assays (Figure S1) indicated that DG-COS NPs reached peak bactericidal efficacy after 10 h of treatment, while HNK/DG-COS NPs achieved optimal activity after 8 h. Live/dead staining (Figure 2C) showed that in the control group, live bacteria exhibited strong green fluorescence and dead bacteria showed weak red fluorescence. However, the green fluorescence intensity was markedly lower in the DG-COS NP and HNK/DG-COS NP groups than in the control and other drug-treated groups, and the red fluorescence was significantly stronger, indicating their potent anti-MRSA effects.

SEM observations (Figure 2D) revealed that MRSA in all treatment groups exhibited morphological changes, with the most pronounced effects being seen in the DG-COS NP and HNK/DG-COS NP groups, including marked wrinkling, rupture, and even structural collapse of the cell membranes. Propidium iodide (PI) staining (Figure 2E) showed that the fluorescence intensity in the DG-COS NP and HNK/DG-COS NP groups was significantly higher than that in the control group (*p* < 0.05). At the same time, the quantitative detection of intracellular component leakage (Figure 2F–H) showed that after co-culture with nanoparticles, the levels of protein, polysaccharide and eDNA in the supernatant were significantly higher than those in the control group (*p* < 0.05). The amount of polysaccharide and eDNA leakage in the HNK/DG-COS NP treatment group was significantly higher than that in the DG-COS NP group (*p* < 0.05). The above results further confirmed that the nanoparticles could effectively destroy the integrity of the MRSA biofilm and induce substantial leakage of intracellular contents.

### 3.3. In Vitro Anti-Biofilm Activity

**Figure 3:**
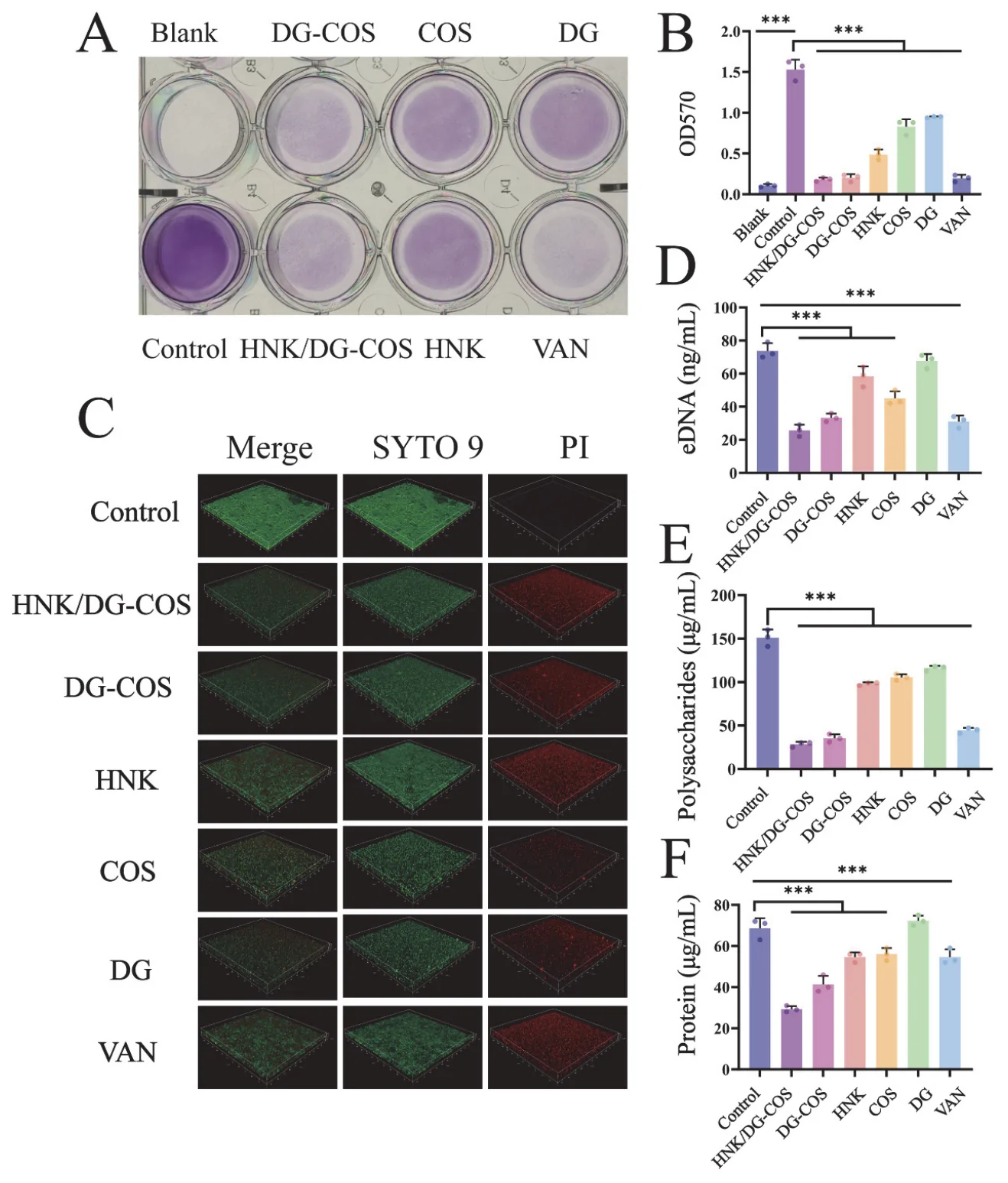
In vitro inhibition of biofilm formation activity of HNK/DG-COS NPs. (**A**) Crystal violet staining of MRSA biofilms following incubation with DG, COS, HNK, DG-COS NPs, and HNK/DG-COS NPs. (**B**) Absorbance at OD_570_ of crystal violet dissolved in acetic acid after MRSA biofilm incubation with DG, COS, HNK, DG-COS NPs, and HNK/DG-COS NPs (*n* = 3). (**C**) Three-dimensional CLSM images of MRSA biofilms after incubation with DG, COS, HNK, DG-COS NPs, and HNK/DG-COS NPs (SYTO 9 indicates live bacteria, and PI indicates dead bacteria). (**D**–**F**) Residual amounts of eDNA, polysaccharides, and protein in MRSA biofilms following treatment with DG, COS, HNK, DG-COS NPs, and HNK/DG-COS NPs (*n* = 3). Data are presented as means ± SD. One-way ANOVA followed by Tukey’s honest significant difference (HSD) test was used for multiple comparisons. *** *p* < 0.001.

The inhibitory effect on biofilm formation was evaluated by crystal violet staining (Figure 3A). The staining intensity in the DG-COS NP and HNK/DG-COS NP groups was substantially lower than that in the other groups. Consistently, the OD_570_ values of these two groups were significantly decreased compared with the control group (*p* < 0.05) (Figure 3B). Confocal laser scanning microscopy (CLSM) of live/dead-stained biofilms (Figure 3C) revealed stronger red fluorescence (indicating dead bacteria) in the DG-COS NP and HNK/DG-COS NP treatment groups relative to the other groups. Analysis of extracellular polymeric substance (EPS) components (Figure 3D–F) demonstrated that the levels of proteins, polysaccharides, and eDNA within biofilms were significantly reduced following DG-COS NP and HNK/DG-COS NP treatments compared with the control group (*p* < 0.05); between these groups, the HNK/DG-COS NP group exhibited the lowest EPS content. Collectively, these observations verify that HNK/DG-COS NPs exert potent inhibitory activity against biofilm formation.

### 3.4. Anti-Inflammation and Cell Migration

**Figure 4:**
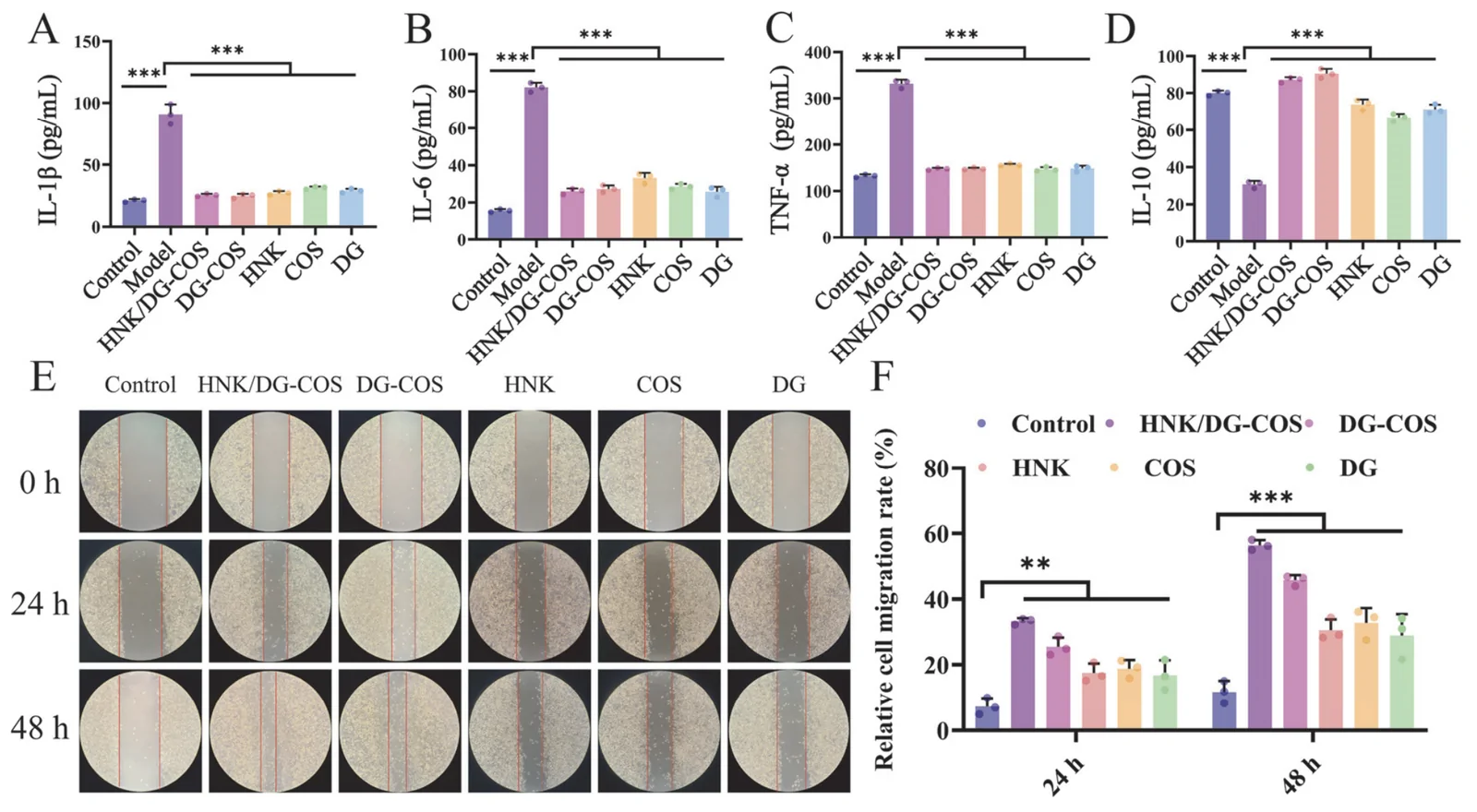
In vitro anti-inflammatory and cell migration effects of HNK/DG-COS NPs. (**A**–**D**) Levels of IL-1β, IL-6, TNF-α, and IL-10 in LPS-stimulated RAW264.7 macrophages after treatment with DG, COS, HNK, DG-COS NPs, and HNK/DG-COS NPs (*n* = 3). (**E**) Representative images of L929 cell migration at 24 h and 48 h after treatment with DG, COS, HNK, DG-COS NPs, and HNK/DG-COS NPs. (**F**) Migration rates of L929 cells at 24 h and 48 h after treatment with DG, COS, HNK, DG-COS NPs, and HNK/DG-COS NPs (*n* = 3). Data are presented as means ± SD. One-way ANOVA followed by Tukey’s honest significant difference (HSD) test was used for multiple comparisons. ** *p* < 0.01, and *** *p* < 0.001.

In LPS-stimulated RAW264.7 cells, treatment with DG-COS NPs and HNK/DG-COS NPs significantly reversed the inflammatory imbalance, wherein the levels of the pro-inflammatory cytokines IL-1β (Figure 4A), IL-6 (Figure 4B) and TNF-α (Figure 4C) were markedly suppressed, while the anti-inflammatory cytokine IL-10 (Figure 4D) was significantly upregulated (*p* < 0.05). The cell migration assay (Figure 4E,F) showed that nanoparticle treatment significantly enhanced cell migration capacity compared with the control group. At 12 h post-intervention, the migration distance was already greater, and by 24 h, the enhancing effect further intensified, with migration rates exhibiting a time-dependent increase (*p* < 0.05).

### 3.5. Safety Evaluation

**Figure 5:**
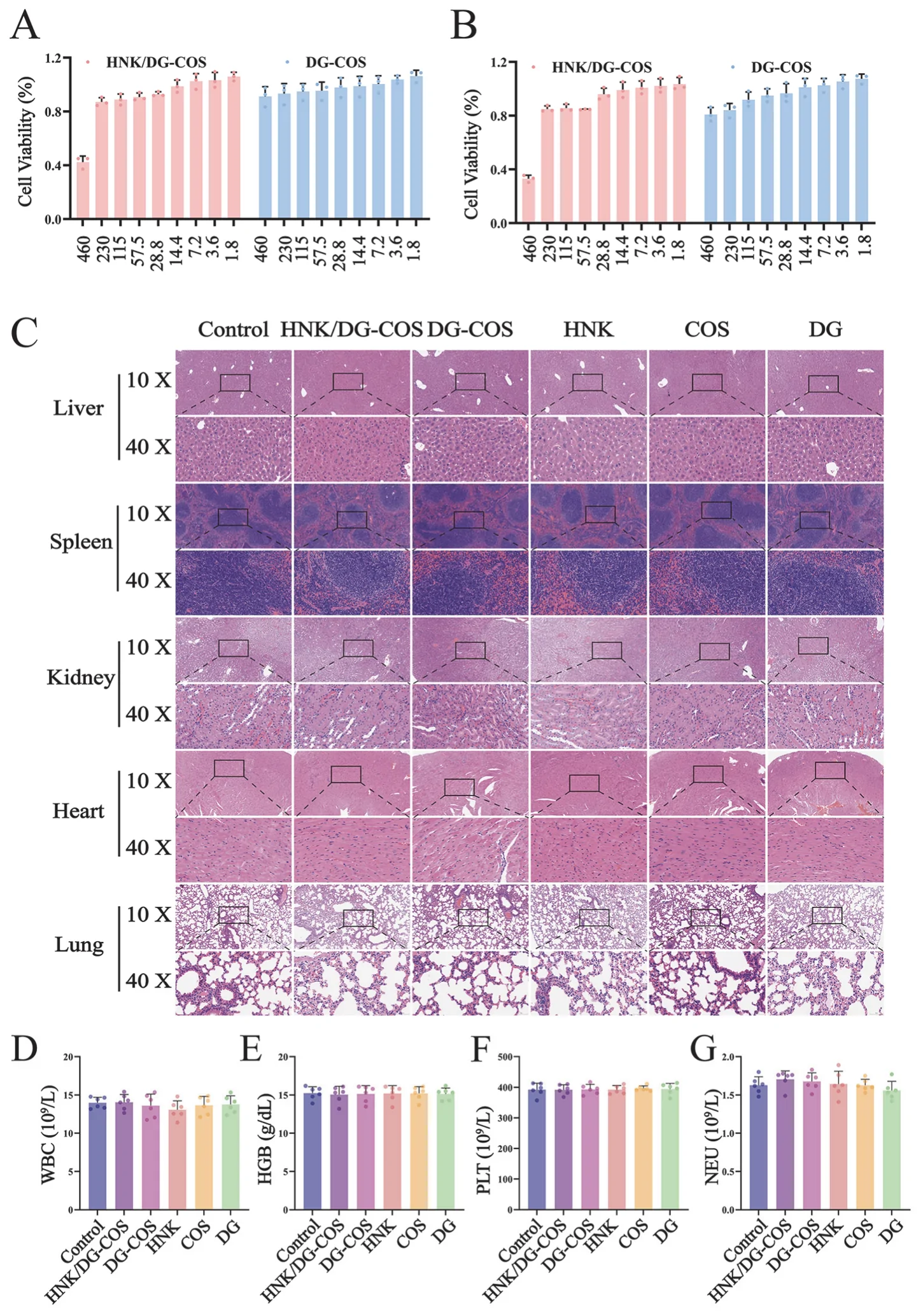
Safety evaluation of HNK/DG-COS NPs. (**A**) Viability of L929 cells following treatment with various concentrations (μg/mL) of DG-COS NPs and HNK/DG-COS NPs for 24 h (*n* = 3). (**B**) Viability of RAW264.7 cells following treatment with various concentrations (μg/mL) of DG-COS NPs and HNK/DG-COS NPs for 24 h (*n* = 3). (**C**) H&E staining of liver, kidney, spleen, heart, and lung tissues from mice 7 days after intraperitoneal injection with DG, COS, HNK, DG-COS NPs, and HNK/DG-COS NPs. The scale bar is 200 μm for the 10X image and 50 μm for the 40X image. (**D**–**G**) Routine blood parameters (WBC, HGB, PLT, and NEU) in mice 7 days after intraperitoneal injection with DG, COS, HNK, DG-COS NPs, and HNK/DG-COS NPs (*n* = 6).

CCK-8 assays (Figure 5A,B and Figure S2) demonstrated that HNK/DG-COS NPs exhibited no significant cytotoxicity towards L929 or RAW cells at most tested concentrations, except for a slight inhibitory effect at the two highest concentrations (460 μg/mL and 230 μg/mL). Live/dead cell staining (Figure S3) confirmed that cells remained in good condition after nanoparticle treatment, with no significant apoptosis or necrosis.

The H&E staining of major organs (Figure 5C) from mice treated with HNK/DG-COS NPs showed no obvious inflammatory infiltration, tissue degeneration, or pathological damage. Hematological parameters (Figure 5D–G), including white blood cells (WBC), hemoglobin (HGB), platelets (PLT), and neutrophils (NEU), exhibited no significant statistical differences between the nanoparticle group and the control group, demonstrating the good safety of HNK/DG-COS NPs in vivo.

### 3.6. In Vivo Wound Healing

**Figure 6:**
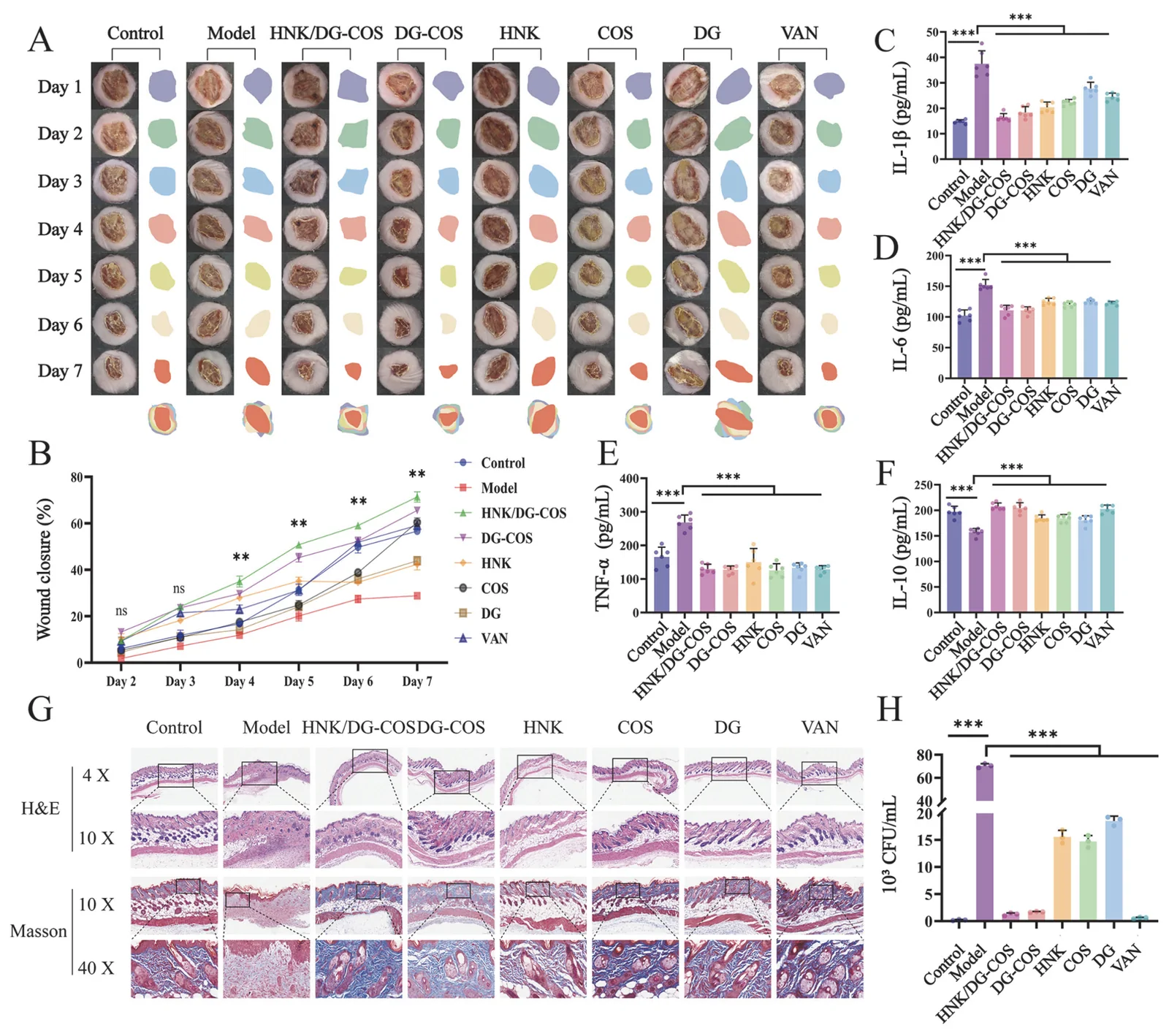
In vivo wound-healing efficacy of HNK/DG-COS NPs. (**A**) Representative photographs and schematic illustrations of mouse skin wound healing after 7 days of treatment with DG, COS, HNK, DG-COS NPs, and HNK/DG-COS NPs. (**B**) Wound-healing rates in mice (*n* = 3). ** indicates that the wound closure rate of the HNK/DG-COS NP group is significantly higher than that of the DG-COS NP group (*p* < 0.01). (**C**–**F**) Serum levels of IL-1β, IL-6, TNF-α, and IL-10 in mice (*n* = 6). (**G**) H&E and Masson’s trichrome staining of wound skin tissue after treatment. Thank you for your reminder. We have added the scale bars to the figure caption. For HE staining, the scale bar is 400 μm for the 4X image and 200 μm for the 10X image. For Masson staining, the scale bar is 200 μm for the 10X image and 50 μm for the 40X image. (**H**) Bacterial load in wound skin tissue after treatment (*n* = 3). Data are presented as means ± SD. One-way ANOVA followed by Tukey’s honest significant difference (HSD) test was used for multiple comparisons. ** *p* < 0.01, and *** *p* < 0.001. The time-dependent wound closure rates were statistically analyzed using two-way repeated-measures ANOVA.

In a mouse model of MRSA-infected skin wounds, wound closure kinetics (Figure 6A,B) showed that the model group had the slowest healing rate throughout the observation period (days 2–7). All treatment groups accelerated wound closure in a time-dependent manner, with the HNK/DG-COS NP and DG-COS NP groups showing the most pronounced effects. During the treatment period from day 4 to day 7, the wound-healing rate of HNK/DG-COS NPs was significantly better than that of DG-COS NPs (*p* < 0.01). Serum levels of inflammatory cytokines (Figure 6C–F) indicated that in the model group, IL-6, IL-1β and TNF-α were significantly elevated, while IL-10 was suppressed. Treatment with HNK/DG-COS NPs significantly reversed this pattern, downregulating IL-6, IL-1β and TNF-α and upregulating IL-10, demonstrating their remarkable anti-inflammatory effect.

The H&E staining of skin tissues (Figure 6G) showed that there were severe disturbances of epidermis integrity with considerable inflammation, edema and tissue disorganization in the model group, while the DG-COS NP and HNK/DG-COS NP groups had almost recovered epidermis integrity, much lower inflammation levels, and a more orderly arrangement of fibroblasts, similar to the normal control group. In addition, Masson’s trichrome staining (Figure 6G) further showed that collagen fiber deposition was sparse and disorganized in the model group, whereas the nanoparticle-treated groups exhibited dense, organized, and fasciculate collagen fibers with significantly increased deposition. The above results indicate that HNK/DG-COS NPs exerted remarkable tissue repair effects.

The bacterial load testing of wound tissue (Figure 6H and Figure S4) demonstrated that the bacterial burden in the model group was markedly elevated, whereas it was significantly reduced in the HNK/DG-COS NP group, with significantly superior anti-MRSA efficacy compared with the other treatment groups.

## 4. Discussion

In this study, we developed honokiol-loaded nanoparticles using two natural polysaccharides as carriers for MRSA-infected wound therapy. By integrating the synergistic antibacterial activity of COS and HNK with the anti-inflammatory and tissue regenerative capacities of DG, the resulting nanoparticles displayed outstanding antibacterial, anti-inflammatory and tissue repair effects. Compared with our previously reported HNK-loaded rhamnolipid-modified chitosan nanoparticles, HNK/DG-COS NPs offer a safer preparation approach without the use of glutaraldehyde. Also, by integrating the anti-inflammatory and tissue regeneration capacities of DG, this nanoplatform enables comprehensive regulatation of the full pathological processes in MRSA-infected wounds.

Under acidic conditions, chitosan carries abundant protonated amino groups (-NH_3_^+^), while β-D-glucan remains electrically neutral or exhibits a weak negative charge due to trace residual impurities. The spontaneous co-assembly of these two polymers into nanoparticle cores is primarily driven by intermolecular hydrogen bonding, with additional contributions from weak electrostatic interactions. Subsequent introduction of sodium tripolyphosphate as an ionic crosslinking agent leads to ionic crosslinking between its polyanionic species and the cationic amino groups of chitosan, thereby significantly enhancing the structural stability of the nanoparticles. The physicochemical characterization collectively confirmed the successful fabrication of DG-COS NPs and efficient encapsulation of HNK. The increase in particle size and zeta potential after drug loading, along with the high encapsulation efficiency, suggests that honokiol was effectively incorporated into the polysaccharide matrix. Compared with DG-COS NPs, HNK/DG-COS NPs exhibited elevated zeta potential. This phenomenon could be explained by HNK-induced conformational rearrangement of COS chains. Hydrogen bonding and hydrophobic interactions between HNK and COS facilitated the exposure of more originally buried protonated amino groups to the particle–water interface. Besides, the loaded HNK might shield residual anionic groups, weakening their charge-neutralizing effect on adjacent protonated amino groups and consequently further increasing the net positive charge density on nanoparticle surfaces. FTIR, Raman, and XRD data consistently indicated that HNK existed in an amorphous, molecularly dispersed state within the nanocarriers, rather than as crystalline aggregates. The disappearance of honokiol’s characteristic crystalline peaks in XRD patterns strongly supports this amorphization, which is beneficial for improving the drug’s aqueous solubility and release behavior.

Results from in vitro antibacterial assays indicated that HNK/DG-COS NPs exhibited potent bactericidal activity against MRSA, which may be attributed to the positively charged surface of the nanoparticles that easily interacts electrostatically with the negatively charged bacterial cell membrane, disrupting the phospholipid bilayer and causing dysfunction of membrane proteins. In the anti-biofilm assays, HNK/DG-COS NPs exerted robust inhibitory effects on the early formation and development of MRSA biofilms through three distinct modes of action. These include the suppression of initial biofilm formation, which was verified by crystal violet staining; the reduction in extracellular polymeric substance accumulation during biofilm maturation; and the elimination of embedded bacteria within developing biofilms, as validated by live/dead staining and CLSM observation. Mechanistically, the chitosan component interferes with bacterial adhesion and biofilm construction via electrostatic interactions and microenvironmental modulation, while HNK further enhances bactericidal efficacy by disrupting bacterial membranes. To comprehensively characterize the biofilm-inhibitory capacity of HNK/DG-COS NPs, we quantitatively analyzed three major EPS components, including eDNA, polysaccharides, and proteins. These constituents play indispensable and distinct structural and physiological roles in biofilm architecture, in that eDNA maintains biofilm mechanical stability and serves as an intercellular adhesion scaffold, polysaccharides support bacterial surface colonization and form a physical barrier that blocks antimicrobial penetration, and proteins promote EPS network crosslinking and correlate closely with bacterial virulence [30]. Quantification of these key EPS components therefore enables a systematic evaluation of biofilm structural damage, rather than merely reflecting the bactericidal activity against planktonic bacteria [31,32]. Consequently, the enhanced activity of HNK/DG-COS NPs can be attributed to synergistic effects of HNK and COS. When treating chronic wound infections, it is crucial to simultaneously disrupt the biofilm and kill the bacteria inhabiting it, as this will minimize the likelihood of bacterial regrowth [33,34,35,36].

Inflammation, which is chronic due to the persistence of the infectious agent, prevents wound healing because it disrupts the functions of keratinocytes and fibroblasts. In this work, in addition to their anti-MRSA effect, the nanoparticles exhibited considerable anti-inflammatory effects on LPS-stimulated macrophages with suppression of pro-inflammatory cytokines (IL-1β, IL-6, and TNF-α) and increased production of the anti-inflammatory IL-10 [37,38]. Such a change in the pro-inflammatory cytokine profile to a pro-resolving one is vital to switching from the inflammatory stage to the proliferative stage of wound healing. Our results revealed that DG-COS NPs exhibited considerable anti-inflammatory activity, indicating that the DG-COS carrier is not biologically inert and can exert auxiliary anti-inflammatory effects. In the present in vivo wound infection model, DG-COS NPs alone were sufficient to alleviate local inflammatory responses, which led to similar therapeutic effects between DG-COS NPs and HNK/DG-COS NPs (Figure 6) and partly obscured the advantages brought by HNK. Free HNK could directly suppress pro-inflammatory mediators, confirming the intrinsic pharmacological activity of HNK. Thus, despite the appreciable anti-inflammatory effects exerted by the blank carrier, HNK still constitutes one of the key contributors to the overall therapeutic performance of these nanoparticles. Furthermore, the increase in cell migration observed in the in vitro experiments confirmed the pro-healing effect of nanoparticles [39]. The capacity of the nanoparticles to regulate inflammation locally, in conjunction with their promotion of cell migration, generates a positive condition for the process of tissue repair.

In a mouse model of infected wounds, the HNK/DG-COS NP-treated group showed a significantly improved healing rate from day 3 to day 7. This healing rate is comparable to that of various other nanomaterial-based treatments for MRSA-infected wounds. For example, Zhao et al. developed a near-infrared-activated Co_7_Fe_3_/ZnO@C nanoenzyme with cascade enzymatic activity [40], whilst Huang et al. developed a AgCINP-based redox hydrogel [41]; both demonstrated excellent efficacy in promoting skin wound healing. HNK/DG-COS NPs exert their effects primarily through three mechanisms. Firstly, HNK/DG-COS NPs eliminate MRSA locally by disrupting its cell membrane. Secondly, the polysaccharide-based carrier creates a favorable environment for the proliferation of cells surrounding the wound. Finally, the reduction in levels of pro-inflammatory cytokines (such as IL-6 and TNF-α) in serum, coupled with an increase in IL-10 levels, indicates that the nanoparticles help to alleviate the inflammatory response induced by the wound and systemically improve the host’s immune status [42,43]. H&E staining results showed that the group treated with HNK/DG-COS NPs exhibited reduced inflammatory cell infiltration and well-organized fibroblast arrangement. In the Masson staining results, the model group displayed lower density of collagen fiber deposition and disordered arrangement. In contrast, in the nanoparticle-treated group, collagen exhibited an ordered fibrillar arrangement [44].

Despite these promising results, we acknowledge that the present study has several limitations. The long-term in vivo toxicity and degradation profile of the nanoparticles have not yet been evaluated. Moreover, the antibacterial assessment was only performed against the Gram-positive pathogen MRSA and was not extended to Gram-negative bacteria. In addition, in vivo experiments targeting mature biofilm eradication were not carried out. To address these shortcomings and further improve the translational potential of these nanoparticles, future work will systematically investigate the inhibitory effects of the nanoparticles against Gram-negative pathogens and explore the underlying molecular mechanisms responsible for bactericidal, anti-inflammatory and tissue-repair activities.

## 5. Conclusions

Taken together, based on two natural polysaccharides, chitosan and β-D-glucan, this study successfully constructed multifunctional HNK nanoparticles with antibacterial and anti-inflammatory activities for the therapy of MRSA-infected wounds. Our primary results indicated that HNK/DG-COS NPs achieved superior antibacterial and anti-biofilm activities by disrupting bacterial cell membranes and dismantling biofilms. HNK/DG-COS NPs significantly mitigated inflammation and accelerated the migration and proliferation of cells in vitro. In an early-stage acute MRSA-infected wound model in mice, HNK/DG-COS NPs were able to eliminate local bacteria, reduce inflammatory damage and promote tissue repair, thereby improving wound condition. In summary, HNK/DG-COS NPs represent a promising antibiotic-free candidate for intervention against early-stage MRSA-infected wounds, based on their demonstrated antibacterial, anti-biofilm, anti-inflammatory, and pro-migratory activities in vitro, as well as their capacity to accelerate wound healing, reduce inflammation, and promote tissue repair in vivo.

## Data Availability

The original contributions presented in this study are included in the article/Supplementary Material. Further inquiries can be directed to the corresponding authors.

## Supplementary Materials

The following supporting information can be downloaded at https://www.mdpi.com/article/10.3390/pharmaceutics18091184/s1: Figure S1. Bacterial colony counts of MRSA treated with DG-COS NPs and HNK/DG-COS NPs for 0, 2, 4, 6, 8, 10, 12, 24, and 48 h. Figure S2. (A) Viability of L929 cells following treatment with various concentrations (μg/mL) of DG-COS NPs and HNK/DG-COS NPs for 12 h (*n* = 3). (B) Viability of RAW264.7 cells following treatment with various concentrations (μg/mL) of DG-COS NPs and HNK/DG-COS NPs for 12 h (*n* = 3). Figure S3. Live/dead staining of L929 and RAW264.7 cells after 12 h treatment with DG, COS, HNK, DG-COS NPs, and HNK/DG-COS NPs. Green fluorescence indicates live cells; red fluorescence indicates dead cells (indicated by white arrows). Scale bars: 50 µm. Figure S4. Bacterial load in wound skin tissue after treatment.
