# Supplementary material for: Honokiol-Loaded β-D-Glucan/Chitosan Nanoparticles with Antibacterial and Anti-Inflammatory Activities for Promoting MRSA-Infected Wound Healing
Source: Pharmaceutics. 2026 Sep 19;18(9):1184. doi: 10.3390/pharmaceutics18091184 (PMC13610103; doi:10.3390/pharmaceutics18091184)
Supplement: Supplementary file 1 [file pharmaceutics-18-01184-s001.zip › pharmaceutics-4504341-supplementary.pdf]

Supporting Information

Honokiol-loaded β-D-Glucan/Chitosan Nanoparticles with Antibacterial and Anti-inflammatory Activities for Promoting MRSA-infected Wound Healing

Shouli Yi [1, 2], Ming Guan[1], Hongjuan Zhang[2], Ping Qiu[2], Shengyi Wang[2,*], Tingjun Hu[1,*]

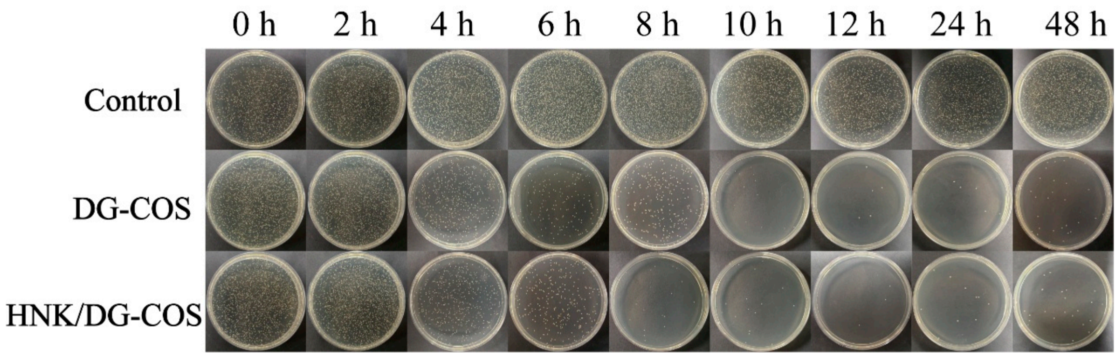

Figure S1. Bacterial colony counts of MRSA treated with DG-COS NPs and HNK/DG-COS NPs for 0, 2, 4, 6, 8, 10, 12, 24, and 48 h.

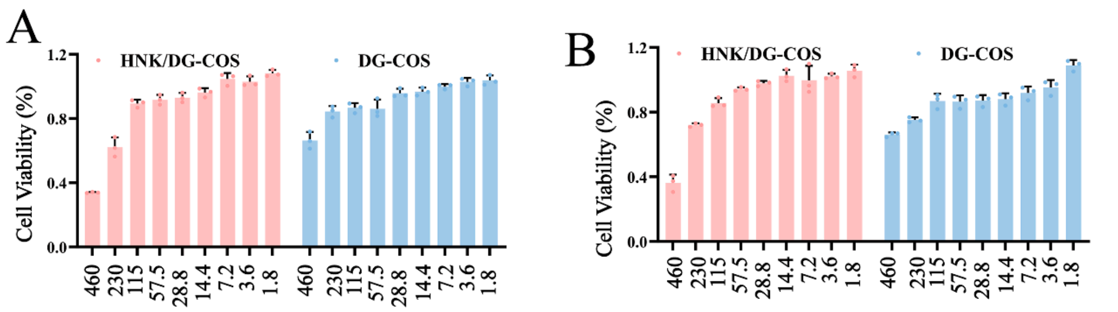

Figure S2. (A) Viability of L929 cells following treatment with various concentrations (μg/mL) of DG-COS NPs and HNK/DG-COS NPs for 12 hours (n = 3). (B) Viability of RAW264.7 cells following treatment with various concentrations (μg/mL) of DG-COS NPs and HNK/DG-COS NPs for 12 hours (n = 3).

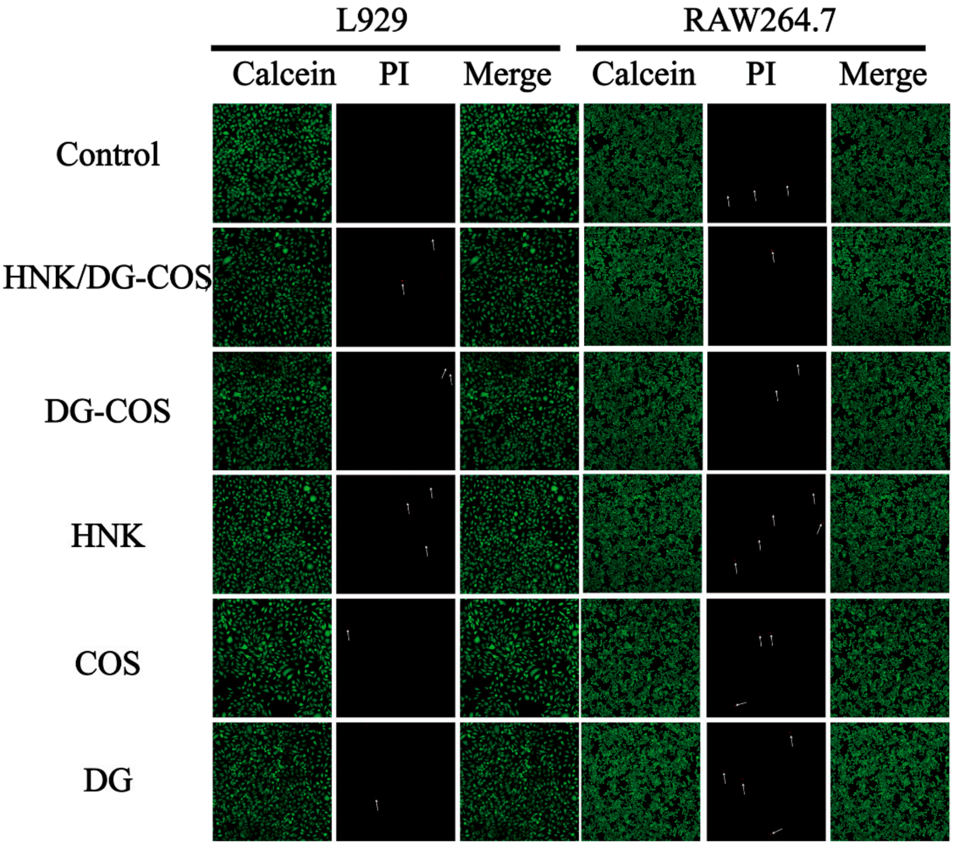

Figure S3. Live/dead staining of L929 and RAW264.7 cells after 12 h treatment with DG, COS, HNK, DG-COS NPs, and HNK/DG-COS NPs. Green fluorescence indicates live cells; red fluorescence indicates dead cells (indicated by white arrows). Scale bars: 50 μm.

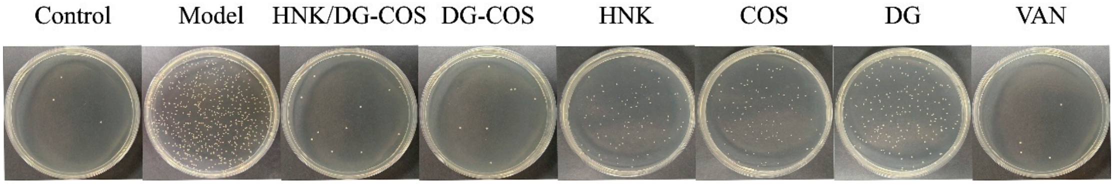

Figure S4. Bacterial load in wound skin tissue after treatment.
